# Low-cost Videolaryngoscope in Response to COVID-19 Pandemic

**DOI:** 10.5811/westjem.2020.5.47831

**Published:** 2020-05-22

**Authors:** Jutamas Saoraya, Khrongwong Musikatavorn, Jariya Sereeyotin

**Affiliations:** * Chulalongkorn University, Faculty of Medicine, Division of Academic Affairs, Bangkok, Thailand; † King Chulalongkorn Memorial Hospital, Department of Emergency Medicine, The Thai Red Cross Society, Bangkok, Thailand; ‡ Chulalongkorn University, Faculty of Medicine, Department of Medicine, Bangkok, Thailand; § Chulalongkorn University, Faculty of Medicine, Bangkok, Thailand; ¶ King Chulalongkorn Memorial Hospital, Department of Anesthesiology, Division of Critical Care,The Thai Red Cross Society, Bangkok, Thailand

## To the Editor

As the novel coronavirus 2019 (COVID-19) has rapidly become a global pandemic, emergency physicians worldwide play essential roles in the frontline management of critically ill patients with COVID-19. In emergency airway management, video laryngoscopes (VL) are recommended over direct laryngoscopy to minimize healthcare worker exposure to aerosolized particles.^1^ However, the VL may be too expensive or unavailable in resource-limited settings, where it is needed to protect the limited number of healthcare providers. We, therefore, reintroduce the idea of creating a low-cost VL from the direct laryngoscope (DL) and a low-cost (approximately $8) smartphone borescope, which is widely available to purchase online. The borescope camera should be secured at the same level as the light sources of the Macintosh blade for the optimal view (Figure, Video). Previous studies of such “Do-It-Yourself” (DIY) VL demonstrated an improved glottic view and increased ease of use in simulated settings for novices and may be comparable to the commercial VL for experienced intubators.^2^,^3^ Moreover, if the capability exists, the disposable blade could be produced from 3D printing.^2^

Emphasis should be on proper training with the DIY VL, as intubation with VL requires different skills when compared with DL and commercial VL.^4^,^5^ Our experience with the DIY VL has led to the following observations. First, we found the device was easy to use, even by novices. Importantly, instead of connecting to a smartphone, the device should be joined with a tablet to provide a larger screen to facilitate visualization. Second, since the borescope has a cylindrical shape, it easily rotates, so the camera should be aligned correctly and tightly secured. If the camera is misaligned or rotated during intubation, the laryngoscopic view on the screen will be oblique or even turned upside down, which may lead to an unsuccessful intubation attempt. Lastly, we noted that the borescope functioned well after it was thoroughly cleaned with detergent and water and disinfected with ortho-phthalaldehyde, our general disinfection protocol. However, if there is a potential concern about contamination or provider safety, the borescope can be discarded as a single-use apparatus since the cost is affordable.

In conclusion, we believe DIY VL is an acceptable option in clinical settings with limited resources in response to emergency endotracheal intubation in the COVID-19 pandemic.

## Supplementary Information

Video The video shows a view from the low-cost videolaryngoscope during simulated intubation. Please see Supplementary File.

## Figures and Tables

**Figure:**
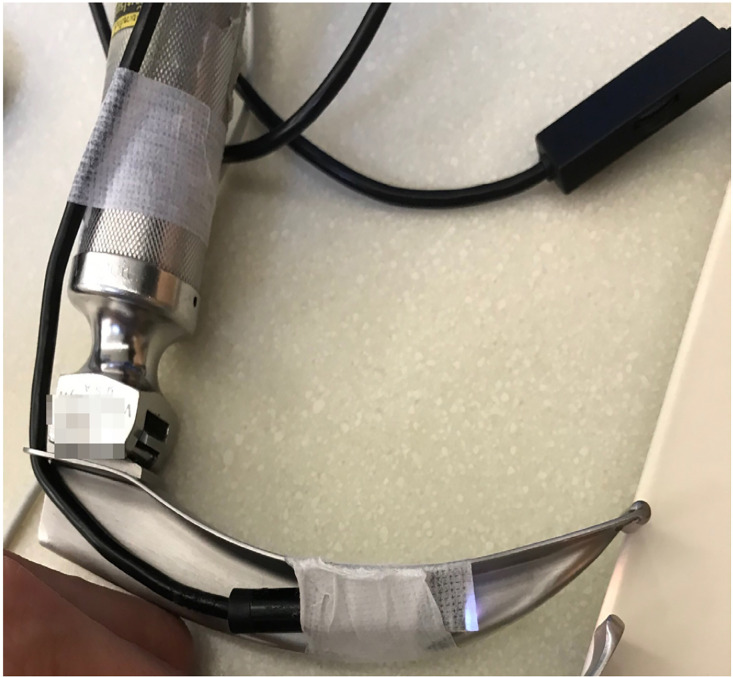
A low-cost videolaryngoscope created from the direct laryngoscope and a low-cost smartphone borescope.
